# Pulmonary Neuroendocrine Cell Hyperplasia Associated with Surfactant Protein C Gene Mutation

**DOI:** 10.1155/2017/9541419

**Published:** 2017-11-09

**Authors:** Norlalak Jiramethee, David Erasmus, Lawrence Nogee, Andras Khoor

**Affiliations:** ^1^Department of Pulmonary Medicine, Mayo Clinic, Jacksonville, FL, USA; ^2^Department of Transplant Medicine, Mayo Clinic, Jacksonville, FL, USA; ^3^Department of Laboratory Medicine and Pathology, Mayo Clinic, Jacksonville, FL, USA; ^4^Eudowood Neonatal Pulmonary Division, Department of Pediatrics, Johns Hopkins University, Baltimore, MD, USA

## Abstract

Familial interstitial lung disease (ILD) is defined as presence of ILD in 2 or more family members. Surfactant protein C* (SFTPC)* gene mutations are rare, but well-known cause of familial ILD. We reported a 20-year-old male, who was referred for lung transplantation. He was symptomatic at age 3 and underwent surgical lung biopsy at age 6, which revealed a nonspecific interstitial pneumonia (NSIP) pattern. Genetic workup revealed a novel* SFTPC* mutation in the first intron with a C to A transversion. At age 21, he underwent bilateral lung transplantation. Explanted lung histology suggested NSIP. In addition there was pulmonary neuroendocrine cell (PNEC) hyperplasia and carcinoid tumorlets. His mother had undergone lung transplantation several years earlier, and her explanted lung showed similar pathology.* SFTPC* mutations are inherited in an autosomal dominant pattern. Various types of ILD have been associated with* SFTPC* mutation including NSIP, usual interstitial pneumonia (UIP), and desquamative interstitial pneumonia (DIP). PNEC hyperplasia has been described to occur in association with lung inflammation but has not been previously described with familial ILD associated with* SFTPC* mutation.

## 1. Introduction

Familial form of ILD is a rare entity. Even among the most common forms of idiopathic ILD, that is, idiopathic pulmonary fibrosis (IPF), the familial form was reported only between 0.5 and 3.7% [1, 2].* SFTPC* mutations are a known cause of familial ILD, which can manifest as varieties of ILD pattern and may occur during childhood or adulthood. We described mother and son with the same* SFTPC* mutation. Both underwent lung transplantation and explanted lung showed similar histology of NSIP, PNEC hyperplasia, and carcinoid tumorlets.

## 2. Case Report

A 20-year-old male was referred to our center for lung transplant evaluation. He was born a full-term infant without any perinatal complications. He started to develop cough at age 3, which was persistent and progressive. He was treated at a tertiary children hospital. Computed tomography (CT) of chest was done when he was 5, which was consistent with “ILD.” After extensive workup without clear diagnosis, he underwent a surgical biopsy of the left lung at the age of 6. This revealed chronic bronchiolitis with bronchiolectasis and patchy interstitial fibrosis. There were no granulomas, pulmonary neuroendocrine cell hyperplasia, or carcinoid tumorlets. The overall pattern was suggestive of nonspecific interstitial pneumonia (NSIP). He underwent genetic testing and was found to have a novel mutation of* SFTPC* with a C to A transversion in the first intron (c.42+333 C>A). He was treated with pulsed methylprednisolone 500 mg intravenously for 2 consecutive days each month. He also received hydroxychloroquine, but this was discontinued after 8 years when he developed visual field defects. He had failure to thrive and required percutaneous gastrostomy tube insertion. He required oxygen at 2-liter/minute flow nasal cannula. He subsequently developed pulmonary hypertension when he was 16 with echocardiographic evidence of biventricular dysfunction. Right heart catheterization showed pulmonary artery pressures (systolic/diastolic/mean) of 52/14/33 mmHg and a pulmonary artery occlusion pressure of 6. He was started on treatment with sildenafil 20 mg orally thrice daily.

He had no other significant medical problems. However, his mother had a history of “pulmonary fibrosis” and had undergone bilateral lung transplantation at a different center at the age of 48 but required another bilateral lung transplantation 3 years later due to chronic lung allograft dysfunction. His mother also had genetic testing after the mutation was found in our patient, and she was found to have the same heterozygous mutation of* SFTPC*. There was no additional family history of lung disease.

**Figure 1:**
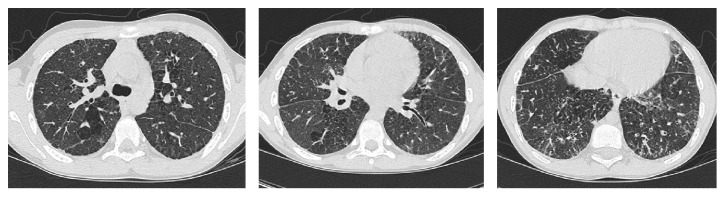
CT of chest showed area of emphysematous changes, several small lung cysts, patchy ground-glass opacity, interlobular septal thickening, and bronchiectasis.

**Figure 2:**
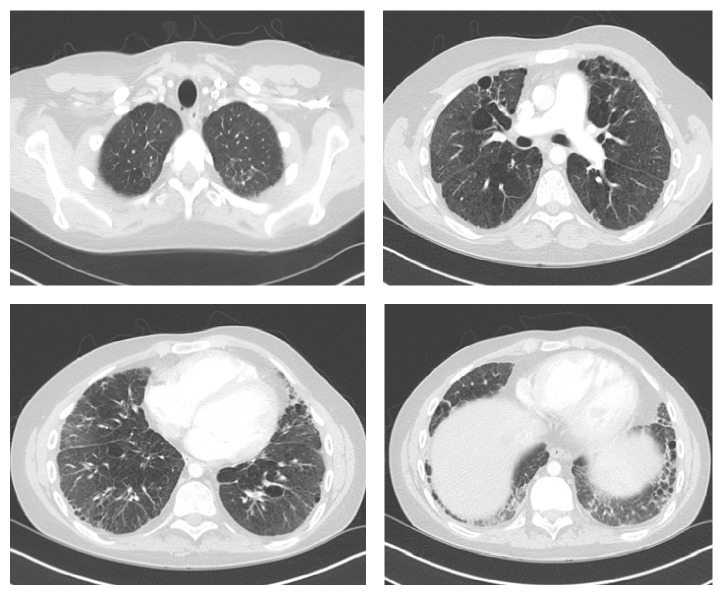
CT of chest showed progressive fibrosis, with increased numbers of lung small cysts, as well as area of emphysema. Additional finding included subpleural honeycombing and mosaic attenuation.

He was referred to our center at the age of 20 when lung transplantation was deemed necessary. Pulmonary function testing (PFT) performed during his transplant evaluation showed mixed obstructive and restrictive impairment, significant air-trapping, and reduced diffusing capacity. FEV1/FVC ratio was 43; FEV1 0.74 L (14% predicted); FVC 1.71 L (27% predicted); TLC 5.21 L (69% predicted); DLCO 6.3 mL/min/mmHg (16%). Six-minute walk test showed total distance of 188 meters and oxygen saturation dropping to 80% on exertion with room air. CT of chest performed at age 12 and age 21 are shown in Figures 1 and 2, respectively.

Echocardiogram (ECHO) performed during pretransplant evaluation showed left ventricular ejection fraction (LVEF) of 32%, mild right ventricular (RV) enlargement, severe decrease in RV systolic function, and a D-shaped ventricle.

He eventually underwent double lung transplantation after being listed for 6 months. ECHO performed on postoperative day 6 showed LVEF of 62%, normal RV size, and systolic function, with resolution of pulmonary hypertension. He had a complicated hospital course due to primary graft dysfunction grade 2, complicated pleural effusion which required redo thoracotomy for downsizing wedge resection, and decortication but was eventually discharged home 7 weeks after transplant and has been thriving 2 years after transplantation.

**Figure 3:**
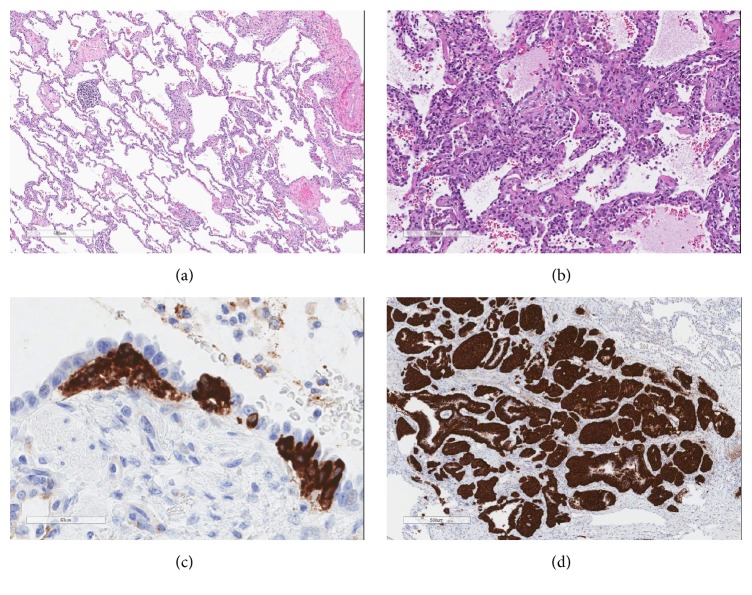
(a) Hematoxylin and eosin (H&E) stain 4x showed chronic interstitial pneumonia, overall pattern most consistently with NSIP. (b) H&E stain 10x showed alveolar septal thickening. (c) Synaptophysin 4x showed PNEC hyperplasia. (d) Synaptophysin 10x showed a carcinoid tumorlet sized approximately 2.5–3 mm.

Histologic examination of the explanted lungs revealed fibrosing chronic interstitial pneumonia with an NSIP pattern and diffuse neuroendocrine cell hyperplasia with multiple carcinoid tumorlets (Figure 3). Immunohistochemical studies for synaptophysin confirmed the presence of PNEC hyperplasia and carcinoid tumorlets.

**Figure 4:**
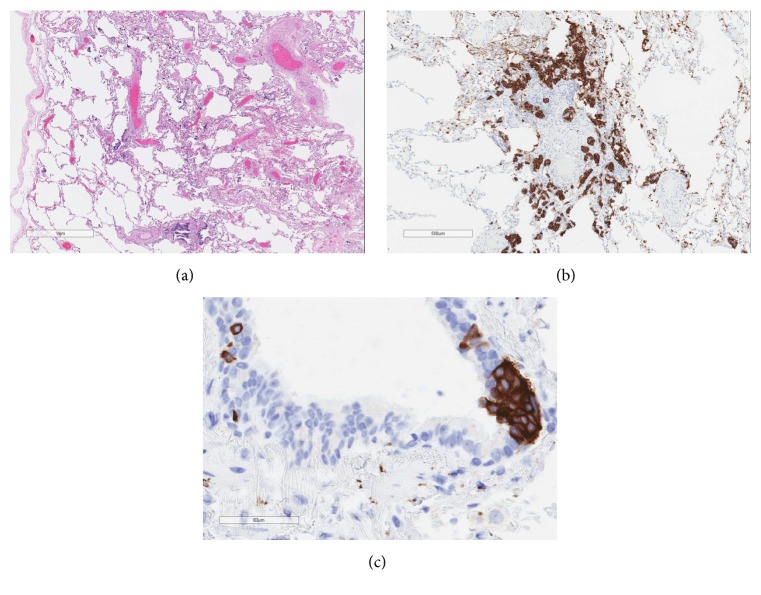
(a) H&E stain 2x showed chronic interstitial pneumonia with alveolar septal thickening and fibrosis, pattern most suggestive of NSIP. (b) Synaptophysin stain 4x showed a carcinoid tumorlet. (c) Synaptophysin stain 40x showed PNEC hyperplasia.

Histologic sections from his mother's lung were also reviewed at our institution. They demonstrated a similar pattern of fibrosing NSIP, PNEC hyperplasia, and multiple carcinoid tumorlets, without presence of any granulomas (Figure 4).

PNEC hyperplasia was diffuse in all explanted lungs (mother and son, right and left). At least 2 hematoxylin and eosin stained sections were examined from each lobe (approximately 2.0 cm^2^ each). Immunohistochemistry for synaptophysin was performed on multiple sections from each patient. In each section examined, the majority of bronchioles (>70%) contained at least one cluster of 5 or more neuroendocrine cells. In addition, each section contained at least one carcinoid tumorlets measuring between 0.2 and 0.5 cm in greatest dimension. Carcinoid tumor (measuring > 0.5 cm in greatest dimension) was not identified.

## 3. Discussion

Familial interstitial pneumonia is generally defined as ILD in 2 or more family members [3, 4]. Over the past decade, there has been increasing knowledge on surfactant protein dysfunction related to familial forms of ILD that can manifest as adult ILD.

Pulmonary surfactant comprised 90% lipids and 10% proteins, which includes surfactant protein- (SP-) A, SP-B, SP-C, and SP-D, with main function to prevent end-expiratory alveolar collapse [5]. Mutations in* SFTPC* can lead to chronic ILD in both adults and children [6–8]. Known mutations of* SFTPC* are transmitted in an autosomal dominant pattern with variable penetrance [4, 9] but can also occur de novo without known mutations in other family members [7, 10, 11].

*SFTPC* is located on chromosome 8 and encodes a 197-amino-acid precursor protein, which undergoes posttranslational processing to the mature SP-C protein of 35 amino acids [5, 12]. Abnormalities that have been reported with* SFTPC* mutations have included usual interstitial pneumonia (UIP), nonspecific interstitial pneumonia (NSIP), desquamative interstitial pneumonia (DIP), chronic pneumonitis of infancy (CPI) [4, 10, 13–15], and pulmonary alveolar proteinosis (PAP) [7, 16]. There have also been reports of individuals in the same family that possessed the genetic mutation, but without any evidence of lung disease [4, 8]. Although multiple mutations have been identified to date, patients with the same mutation have been found to express different phenotypes of lung disease as demonstrated in a study in which all patients had the most frequently identified p.Ile73Thr mutation, but biopsy showed either CPI or DIP [10]. Another study in one family with the p.Leu188Gln mutation showed that some members had NSIP pattern, while several others had a UIP pattern [4]. The reason for this variable expression was speculated to be due to viral infection, modifying gene, and/or environmental factors [17].

Pulmonary neuroendocrine cells (PNEC) play an important role in the developmental process of the lung and therefore seem to be present in greater amount in children's lungs as compared to adults [18, 19]. Hyperplasia of PNEC has been reported to be associated with various underlying lung disease including bronchopulmonary dysplasia, pulmonary hypertension [20, 21], and prolonged mechanical ventilation [22]. PNEC hyperplasia without an underlying pulmonary disease is known as neuroendocrine cell hyperplasia of infancy (NEHI) in children and in adult counterpart is known as diffuse idiopathic neuroendocrine cell hyperplasia (DIPNECH). It has been increasingly recognized but still remains a relatively rare entity [23]. There was no formal criterion for defining excess of pulmonary neuroendocrine cells, but it was proposed in previous study [24] that finding of PNEC within 70% of bronchioles in the lung biopsy specimen and >10% in an individual airway is consistent with diagnosis of NEHI in appropriate clinical setting. This criterion has been questioned in study by Yancheva et al. [25] that children with various lung diseases would also meet this criterion and therefore while the criterion might be sensitive for diagnosis of NEHI, it may lack specificity. They proposed using the average percentage of NEC per airway to identify NEHI more accurately; however, there was still significant overlap in the number of NEC/airway between NEHI and other lung diseases including surfactant protein disorder. On the contrary, in diagnosis of DIPNECH, the same quantitative measurement did not apply since the numbers of NEC in adults are much less compared to children, 4 NEC in 10,000 epithelial cells [26]. DIPNECH was defined more loosely by World Health Organization as PNEC proliferation that may be confined to bronchial or bronchiolar epithelium, extraluminal proliferation in the form of tumorlets, or development of carcinoid tumors [27]. However, the specific quantification was not provided. A study by Marchevsky et al. attempted to provide diagnostic criteria for DIPNECH and concluded that the presence of 5 or more neuroendocrine cells, singly or in clusters located within the basement membrane of bronchiolar epithelium of at least 3 bronchioles, combined with 3 or more carcinoid tumorlets can be used consistently to diagnose DIPNECH [28]. Another study proposed combination of clinical, radiographic, histologic, and serum markers for diagnosis, but of note, they found 100% of DIPNECH patients had presence of carcinoid tumorlets in biopsy sample [23]. DIPNECH has radiographic findings of ground-glass opacity, air-trapping, mosaic attenuation, and histology commonly showing constrictive bronchiolitis [23]. Besides an underlying condition, there are no clear histologic differences between reactive PNEC and DIPNECH [28]. However, DIPNECH is more strongly associated with carcinoid tumors [29].

Various radiographic findings have been reported in association with* SFTPC* mutations. Patterns of combined emphysema and pulmonary fibrosis have been described in children [30] and adults who never smoked [31]. Findings that have consistently been reported in* SFTPC* mutations are ground-glass opacity, multiple lung cysts of varying sizes, and septal thickening [8, 13, 32]. Two different reports described a spectrum of progression from initial diffuse ground-glass opacity to multiple lung cysts and pulmonary fibrosis [11, 30].

In our patient, the pathology of the explanted lung suggested NSIP pattern, emphysematous changes, and pulmonary fibrosis, all of which have previously been described in patients with* SFTPC* mutations. The unexpected finding in this case was the presence of diffuse neuroendocrine cell hyperplasia and carcinoid tumorlets. The number of PNEC and carcinoid tumorlets were numerous and theoretically met the diagnostic criteria of DIPNECH proposed by previous studies as mentioned above [23, 28, 29]. In addition, the quantity of PNEC would also have met proposed criteria for NEHI [24]. However, our patient did not have PNEC hyperplasia in his biopsy at the age of 6 and his mother developed the disease in her adulthood and therefore their clinical manifestation would not be consistent with NEHI. Since our patient and his mother possessed a novel mutation that had not previously been described, it was difficult to determine if the PNEC hyperplasia in our patient represented a reactive process secondary to his ILD, a variant of phenotypic expression of* SFTPC* mutation with common exposure or infection in the family (the finding was also present in his mother who had the same genetic mutation), or a coexistence of a separate primary disease (DIPNECH). His CT of chest finding revealed emphysema, ground-glass opacities, multiple lung cysts, septal thickening, and subpleural honeycombing, which have all been reported to be associated with* SFTPC* mutations. In addition, there was some evidence of air-trapping and mosaic attenuation, which are well-described radiographic findings in DIPNECH [23, 33].

To our knowledge, there has not been familial case of DIPNECH, although there has been familial case of neuroendocrine cell hyperplasia of infancy (NEHI), which was related to mutation of NKX2 encoding TTF-1. TTF-1 regulates the expression of surfactant proteins including SP-C [34]. We speculated that gene related to PNEC hyperplasia such as those seen in NEHI may be closely linked to gene affecting surfactant protein expression and vice versa.

We report a unique situation of a novel mutation in* SFTPC* in a mother and her son, where clinical and pathological correlation can be made due to lung transplantation of both and examination of histological findings of the explanted lungs. The mutation in* SFTPC* occurred in intron 1 with a C to A transversion of both mother and son, but with ILD manifesting earlier in life of the latter. Although the mutation was not identified in the transcribed portions of the genes or the splice junction, and therefore it cannot be certain if it affected protein synthesis, it could conceivably introduce a new site for mRNA splicing. This sequence variant is not listed in the 1000-genome project, indicating that it is a very rare variant. Previous reports of mutation in intron 4 of* SFTPC* have shown that it caused alteration in the splicing site and led to abnormal protein production [9, 35]. Although causality cannot be strongly established, given the same* SFTPC* mutation pattern and same pathology finding of NSIP, PNEC hyperplasia, and carcinoid tumorlets in both mother and son, this suggested possible genetic abnormality could be related to the* SFTPC *mutation or another gene yet to be identified. Currently, there is no other explanation for this rare and unique pathology finding in this family. Our patient underwent successful bilateral lung transplantation with normal lung function two years after transplantation.
